# Alterations of NK Cell Phenotype During Pregnancy in Multiple Sclerosis

**DOI:** 10.3389/fimmu.2022.907994

**Published:** 2022-07-04

**Authors:** Anne Wisgalla, Caren Ramien, Mathias Streitz, Stephan Schlickeiser, Andreea-Roxana Lupu, Anke Diemert, Eva Tolosa, Petra C. Arck, Judith Bellmann-Strobl, Nadja Siebert, Christoph Heesen, Friedemann Paul, Manuel A. Friese, Carmen Infante-Duarte, Stefan M. Gold

**Affiliations:** ^1^ Medizinische Klinik m.S. Psychosomatik, Charité – Universitätsmedizin Berlin, Campus Benjamin Franklin, Berlin, Germany; ^2^ Experimental and Clinical Research Center, Max Delbrück Center for Molecular Medicine in the Helmholtz Association (MDC) and Charité-Universitätsmedizin Berlin, Berlin, Germany; ^3^ Institut für Neuroimmunologie und Multiple Sklerose (INIMS), Universitätsklinikum Hamburg-Eppendorf, Hamburg, Germany; ^4^ Institut für Medizinische Immunologie, Charité – Universitätsmedizin Berlin, Campus Virchow Klinikum, Berlin, Germany; ^5^ BIH Center for Regenerative Therapies (BCRT), Charité – Universitätsmedizin Berlin, Campus Virchow Klinikum, Berlin, Germany; ^6^ Department of Experimental Animal Facilities and Biorisk Management, Friedrich-Loeffler Institut, Federal Research Institute for Animal Health, Greifswald-Insel Riems, Germany; ^7^ Cantacuzino National Military Medical Institute for Research and Development, Bucharest, Romania; ^8^ Klinik für Geburtshilfe und Pränatalmedizin, Universitätsklinikum Hamburg-Eppendorf, Hamburg, Germany; ^9^ Institut für Immunologie, Universitätsklinikum Hamburg-Eppendorf, Hamburg, Germany; ^10^ NeuroCure Clinical Research Center, Charité – Universitätsmedizin Berlin, Campus Charité Mitte, Berlin, Germany; ^11^ Klinik für Psychiatrie und Psychotherapie, Charité – Universitätsmedizin Berlin, Campus Benjamin Franklin, Berlin, Germany

**Keywords:** natural killer cells, multiple sclerosis, pregnancy, immune tolerance, immune profiling, FlowSOM clustering

## Abstract

In multiple sclerosis (MS), relapse rate is decreased by 70-80% in the third trimester of pregnancy. However, the underlying mechanisms driving this effect are poorly understood. Evidence suggests that CD56^bright^ NK cell frequencies increase during pregnancy. Here, we analyze pregnancy-related NK cell shifts in a large longitudinal cohort of pregnant women with and without MS, and provide in-depth phenotyping of NK cells. In healthy pregnancy and pregnancy in MS, peripheral blood NK cells showed significant frequency shifts, notably an increase of CD56^bright^ NK cells and a decrease of CD56^dim^ NK cells toward the third trimester, indicating a general rather than an MS-specific phenomenon of pregnancy. Additional follow-ups in women with MS showed a reversal of NK cell changes postpartum. Moreover, high-dimensional profiling revealed a specific CD56^bright^ subset with receptor expression related to cytotoxicity and cell activity (e.g., CD16^+^ NKp46^high^ NKG2D^high^ NKG2A^high^ phenotype) that may drive the expansion of CD56^bright^ NK cells during pregnancy in MS. Our data confirm that pregnancy promotes pronounced shifts of NK cells toward the regulatory CD56^bright^ population. Although exploratory results on in-depth CD56^bright^ phenotype need to be confirmed in larger studies, our findings suggest an increased regulatory NK activity, thereby potentially contributing to disease amelioration of MS during pregnancy.

## Introduction

During pregnancy, the maternal immune system is regulated to ensure immune tolerance toward fetal alloantigens and maintenance of immune competence against infections (1). Intriguingly, this state of endogenous immunomodulation also affects maternal autoimmunity leading to a reduced activity of various autoimmune disorders during pregnancy (2). In multiple sclerosis (MS), relapses are diminished by 70%-80% in the third trimester followed by increased MS activity postpartum (3, 4).

While clinically the protective effect of pregnancy on MS disease activity is well-established, its underlying biological mechanisms have remained difficult to decipher. In placental mammals, evolutionary pressure on reproduction has driven the development of specific pathways that establish immune tolerance for a successful pregnancy (5, 6). However, explanations of the temporary amelioration of MS during pregnancy have mainly focused on a non-specific general modulation of the maternal immune system, such as shifts in circulating immune cell populations and cytokines [reviewed in (7)], as well as immunomodulatory effects of pregnancy hormones (8, 9). Recently, studies in murine and human pregnancy have indicated that T cell responses are modulated in a clone-specific fashion, suggestive of potentially antigen-specific regulation in healthy pregnancy and autoimmunity (10–13). Identifying regulatory drivers that orchestrate these cell-mediated mechanisms during pregnancy could thus provide crucial insights into the pathways of tolerance induction.

In this context, natural killer cells (NK cells) may serve as an important modulator, as they exert immunoregulatory functions and have been suggested to play a protective role in MS (14). Human NK cells are generally divided into two major populations (15): CD56^dim^ NK cells are regarded as highly cytotoxic effector cells in contact to transformed or virus-infected cells, while CD56^bright^ NK cells constitute the main regulatory subset that can modulate other immune cells through cytokine secretion and direct cytotoxicity. In this line, it has been reported that CD56^bright^ NK cells are able to suppress activated CD4^+^ T cells through cytotoxic killing (16). In MS, this suppressive capacity toward T cells was found to be diminished (17, 18) and temporary NK cell deficits were associated with disease activity and clinical exacerbation (19, 20) suggesting an impaired potential of NK cells to limit T cell driven inflammation in autoimmunity. Importantly, several MS therapies such as daclizumab, dimethyl fumarate or interferon-beta seem to increase frequencies and cytotoxicity of CD56^bright^ NK cells (21–23). It is therefore conceivable that NK cells might also contribute to disease amelioration of MS during pregnancy. Indeed, evidence suggests a shift in NK cell frequencies, with an increase of CD56^bright^ NK cells and a decrease of CD56^dim^ NK cells from the first to the third trimester that coincided with the reduced MS activity during that period (24). However, these longitudinal findings (24) are limited by the small sample size. More recent studies (25–27) all used cross-sectional study designs in small samples, in some cases lacked healthy control groups, and varied in terms of the exact NK subset definitions used. Moreover, studies in this area (24–27) have been restricted to solely enumerating the broad NK cell populations instead of evaluating more specific phenotypic changes.

Here, we aimed to conduct a confirmatory analysis of pregnancy-related NK cell shifts in a large longitudinal cohort of pregnant women with and without MS, and to provide additional exploratory evidence on in-depth phenotype modulations of NK cells in MS. Investigating the role of NK cells during pregnancy in health and MS could support a better understanding of their regulatory capacities with potential relevance for tolerance induction in autoimmunity.

## Materials and Methods

### Subjects and Recruitment

To assess the dynamics of NK cells over the course of pregnancy, women from 3 longitudinal cohorts were included in the present immunophenotyping study. Patients in the Hamburg MS cohort were recruited through the Multiple Sklerose Tagesklinik of the Universitätsklinikum Hamburg- Eppendorf. Patients in the Berlin pregnancy cohort in MS (PreCoMS) were recruited by the Charité NeuroCure Research Center. Healthy pregnant women were recruited as part of the Prenatal Identification of Children’s Health (PRINCE) study at the Klinik für Geburtshilfe und Pränatalmedizin, Universitätsklinikum Hamburg-Eppendorf. The study was approved by the local ethics committees (Ethikkommission der Ärztekammer Hamburg; PV3558, PV3694; Ethikkommission der Charité – Universitätsmedizin Berlin, EA1/173/13) and all participants gave written informed consent prior to enrolment. Participants were examined in the respective medical centers by a neurologist or gynecologist (PRINCE) at three timepoints during pregnancy (Tri 1: gestational week 8-14; Tri 2: gestational week 20-24, Tri 3: gestational week 30-36). MS patients were additionally seen after child-birth: 3 months postpartum in Hamburg and 3, 6, 9 and 12 months postpartum in Berlin. During clinical visits, blood was sampled and a neurological/gynecological exam was performed. For analysis, all subjects with complete flow cytometry data within pregnancy and at 3 months postpartum (for MS patients) were selected. Data for the healthy cohort shown here refer to women without autoimmune diseases, however, the same pattern was observed if women with any chronic disease were excluded, such as hypo-/hyperthyroidism (17/180 women) and asthma/emphysema (9/180 women). Details of demographic data and descriptors of MS severity are listed in **Table 1**.

**Table 1:** Demographics and MS descriptors.

	Healthy cohort Hamburg	MS cohort Hamburg	MS cohort Berlin
Participants (n)	180	24	8
Age (years, mean; SD)	31.1; 3.7	30.2; 4.8	32; 3.7
Mothers pregnant with first child (n; %)	115; 64%	21; 88%	6; 75%
Sex of the child (n, boy/girl/n.d.)	103/77/0	10/11/3	5/3/0
Disease course (n; RRMS/CIS/other)	n.a.	20/3/1	7/1/0
Disease duration (years, mean; SD)	n.a.	2.9; 2.5	5.5; 3.8
EDSS at Tri 1 (score, median; IQR)	n.a.	1.0; 2.0	1.5; 0.1
EDSS at PP 3 (score, median; IQR)	n.a.	1.0; 2.0	0.5; 1.6
EDSS at PP 6 (score, median; IQR)	n.a.	n.d.	0.8; 1.8
Last DMT before pregnancy (n, none/GA/IFNb/DMF/aHST)	n.a.	12/5/5/1/1	3/3/1/1/0
DMT at PP 3 (n, none/GA/IFNb/DMF)	n.a.	18/3/1/1	8/0/0/0
Breastfeeding at PP 3 (n, yes/no/n.d.)	n.a.	18/4/2	7/1/0
Relapse in 2 years prior to pregnancy (n, yes/no/n.d.)	n.a	16/7/1	6/2/0
Relapse in pregnancy (n, yes/no)	n.a.	2/22	0/8
Relapse until PP 3 (n, yes/no)	n.a.	4/20	0/8

aHST, autologous hematopoetic stemcell transplantation; CIS, clinically isolated syndrome; DMF, dimethylfumarate; DMT, disease modifying therapy; EDSS, Expanded Disability Status Scale; GA, glatiramer acetate; IFNb, interferon beta; IQR, interquartile range; n.a., not applicable; n.d., no data; other, patients with suspected primary progressive MS; RRMS, relapsing-remitting multiple sclerosis; SD, standard deviation; PP 3, 3 months postpartum.

### Sample Collection and Flow Cytometry

Peripheral blood of healthy controls and MS patients in Hamburg were sampled into EDTA tubes (Sarstedt, Germany). Thereafter, blood samples were stained freshly in the dark at 4°C with an antibody cocktail containing CD3 AF647 (clone UCHT1), CD4 V500 (clone RPA-T4; BD Biosciences, USA), CD8 APC-Cy7 (clone RPA-T8), CD14 PB (clone HCD14), CD16 FITC (clone 3G8), CD19 PE-Cy7 (clone HIB19), CD45 PerCP-Cy5.5 (clone HI30), CD56 PE (clone HCD56; Biolegend, USA if not stated otherwise). After staining, leukocytes were fixed and erythrocytes lysed with BD FACS Lysing Solution (BD Bioscience, USA), then washed twice in PBS (520 x g, 5 min, 4°C). Subsequently, samples were measured on a FACS Canto II (BD Bioscience, USA).

In the Berlin MS cohort, peripheral blood mononuclear cells (PBMCs) were isolated from heparinized whole blood by density centrifugation (Biochrom GmbH, Germany) and cryopreserved in RPMI-1640 media (Gibco Life Technologies, Germany) with 20% fetal bovine serum (Sigma Aldrich, Germany) and 10% dimethyl sulfoxide (Sigma Aldrich, Germany) in liquid nitrogen. For flow cytometric assessment of NK cells, frozen PBMCs were thawed and washed with RPMI-1640 media with 10% fetal bovine serum. Sample integrity after thawing was determined microscopically with trypan blue, and through cell viability staining with Zombie NIR Fixable Viability Kit (Biolegend, USA). PBMCs were sequentially stained with FcR Blocking Reagent (Miltenyi Biotec, Germany) to minimize unspecific binding of antibodies, and with the following antibodies (Biolegend, USA if not stated otherwise) diluted in PBS with 0.5% BSA (Gibco Life Technologies, Germany/Sigma Aldrich, Germany): CCR7 BV421(clone G043H7), CD127 BV785 (clone A019D5), CD14 Alexa700 (clone 63D3), CD16 BUV395 (clone 3G8; BD Biosciences, USA), CD19 Alexa700 (clone HIB19), CD20 Alexa700 (clone 2H7), CD27 PE (clone M-T271), CD3 Alexa700 (clone UCHT1), CD56 PE/Dazzle594 (clone 5.1H11), CD94 APC (clone DX22), CX3CR1 BV605 (clone 2A9-1; BD Biosciences, USA), DNAM1 PerCP/Cy5.5 (clone 11A8), NKG2A PE/CY7 (clone Z199; Beckman Coulter, USA), NKG2C Alexa488 (clone #134591; R&D Systems, USA), NKG2D BV510 (clone 1D11), NKp46 BV650 (clone 9E2). Antibodies for cell surface staining were incubated in the dark at room temperature for 15 min. Data acquisition was performed on LSR Fortessa (BD Biosciences, USA).

### Magnetic Resonance Imaging (MRI)

Patients in the Berlin MS Cohort were invited to MRI scans for all timepoints outside pregnancy. MRI scans were generally obtained on the same day as blood sampling or at maximum within 8 days. All MRI data were acquired on the same 1.5 Tesla scanner (MAGNETOM Sonata Siemens, Erlangen, Germany) using a volumetric high-resolution T1 weighted magnetization prepared rapid acquisition gradient echo (MPRAGE) sequence (repetition time [TR] = 2,110 ms; echo time [TE] = 4.38 ms; inversion time [TI] = 1100 ms; FOV=256 x 256 mm^2^; slice thickness 1mm) as well as an axial T2 weighted turbo inversion recovery magnitude sequence (TIRM) (TR/TE/TI = 10,000/108/2,500 ms; field of view [FOV] = 256 × 256 mm^2^, slice thickness 3.0 mm). Lesion segmentation for total lesion volume was semi-automatically performed on T2 TIRM images of all patients by MATLAB-based Lesion Segmentation Toolbox (LST) for Statistical Parametric Mapping (28) and checked and verified by three expert raters under the supervision of a board-certified neuroradiologist using ITK-SNAP (www.itksnap.org) (29).

### Data Analysis and Statistics

Flow cytometric data of the Hamburg and Berlin cohorts were first compensated and then manually gated for the major NK cell populations CD56^bright^ and CD56^dim^ using FlowJo Software (Treestar, USA). In both cohorts, CD3^+^ T cells were excluded before selecting NK cells (gating in the Berlin cohort also excluded CD19^+^, CD20^+^, and CD14^+^ cells; for gating strategy, see **Supplementary Figure 1**).

High-dimensional NK cell data from Berlin MS patients were explored by a computational analysis utilizing the unsupervised clustering algorithm FlowSOM (30). FlowSOM assigns each cell in to clusters of phenotypically similar cells using self-organizing maps (SOM), a type of artificial neural network. A second metaclustering step allows for identification of larger clusters resembling biologically relevant cell populations which can subsequentially be analyzed for differential marker expression and changes in cell frequencies. FlowSOM was performed in Cytobank (Cytobank Inc., USA) with clustering of pre-gated Lin^-^ CD56^+^ NK cells based on their expression of 12 surface markers (e.g., CD56, CD16, NKG2C NKp46, DNAM, NKG2D, NKG2A, CD27, CX3CR1, CCR7, CD94, and CD127). SOM clustering was conducted using a 9 X 9 grid, resulting in 81 total clusters that were then grouped into 20 metaclusters by consensus clustering. Metaclusters were visualized in a minimal spanning tree (MST) in which clusters that are close contain phenotypically similar cells (see **Supplementary Figure 2**). For further analysis, cell frequencies of each metacluster were exported and assessed in R for statistically significant frequency changes to identify pregnancy-associated NK cell subsets. Finally, metaclusters exhibiting significant frequency changes were manually characterized for marker expression (i.e., median fluorescence intensity) using MST visualizations conducted by Cytobank and heatmap obtained in R. In the results section of this article, metaclusters are simply referred to as clusters.

Statistics were performed in R version 4.0.5. Differences in cell frequency between timepoints were compared by paired Wilcoxon tests.

## Results

### Pregnancy Induces an Expansion of CD56^bright^ NK Cells in Healthy Women and Women With MS

The three cohorts were comparable with respect to mothers’ age, parity, and sex of the child (see **Table 1**). First, we conducted flow cytometric assessment of the two major NK cell populations, CD56^bright^ and CD56^dim^ NK cells, during the first, second and third trimester of pregnancy in the group of healthy women (n=180, healthy cohort, Hamburg) and in one of the groups of female MS patients (n=24, MS cohort, Hamburg). For MS patients, NK cell populations were additionally measured at three months postpartum.

**Figure 1:**
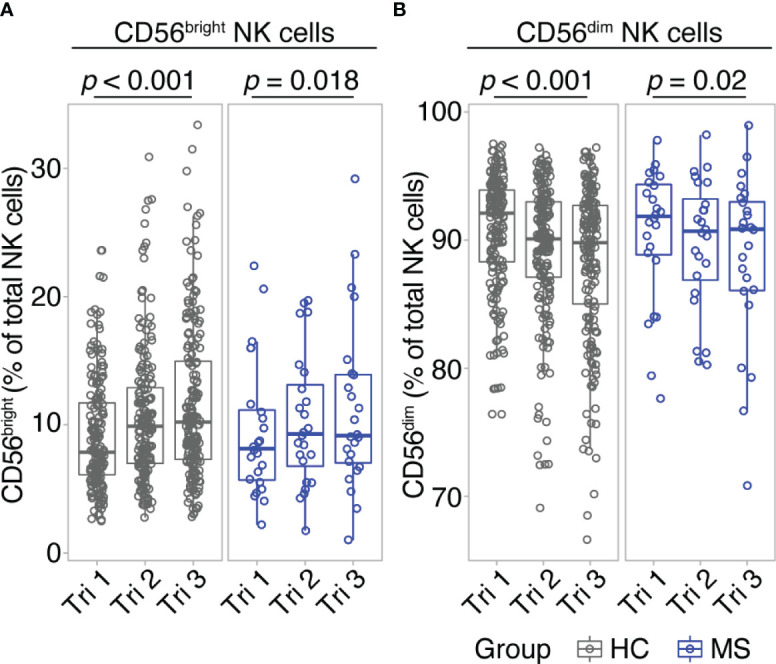
CD56bright NK cells increase throughout pregnancy. Frequency of CD56^bright^ NK cells **(A)** and CD56^dim^ NK cells **(B)** in healthy pregnant women (HC, gray) and pregnant MS patients (MS, blue, Hamburg cohort) shown for each trimester of gestation. Boxplots depict median and inter-quartile range, overlaid with datapoints of individuals. Statistical analysis performed by Wilcoxon paired test between trimesters 1 and 3.

Comparing early vs. late pregnancy, we found a significant increase of CD56^bright^ NK cell frequencies from the first to the third trimester in women with MS as well as in healthy controls (**Figure 1A**). Despite the large difference in sample size, the effect size of this pregnancy-related increase in CD56^bright^ NK cells was comparable for MS (d = -0.54; 95% CI [-0.99, -0.11]) and HC (d = -0.54; 95% CI [-0.7, -0.39]). Mirroring the shifts of the CD56^bright^ population, CD56^dim^ NK cell frequencies decreased toward trimester 3 in MS patients and healthy controls (d = 0.54; 95% CI [0.11, 0.99] and d = 0.54; 95% CI [0.39, 0.7] for MS and HC, respectively, **Figure 1B**). Additional postpartum follow-ups in the MS cohort demonstrated a reversal of pregnancy-associated shifts in the NK cell compartment: CD56^bright^ NK cell frequency was diminished and CD56^dim^ NK cell frequency increased at three months postpartum compared to late-pregnancy (d = 0.51; 95% CI [0.07, 0.96] and d = -0.51; 95% CI [-0.96, -0.07] for CD56^bright^ and CD56^dim^, respectively, **Supplementary Figure 3**). Together, our data illustrate that NK cells undergo pronounced frequency changes during and after pregnancy, in particular pointing toward a pregnancy-induced expansion of regulatory CD56^bright^ NK cells in the third trimester. As comparable results from MS and HC indicate, this shift seems to display a general phenomenon of healthy pregnancy rather than an MS-specific effect.

### In-Depth NK Cell Profiling Shows Alterations of NK Cell Phenotype Related to Cell Activity and Cytotoxicity

**Figure 2:**
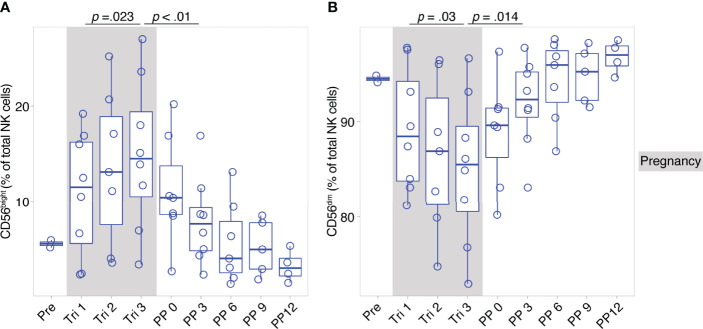
Pregnancy-induced shifts of NK cells throughout and after gestation in Berlin MS cohort. Frequency of CD56^bright^ NK cells **(A)** and CD56^dim^ NK cells **(B)** in pregnant MS patients (MS, Berlin cohort) shown for each trimester of gestation and 1 year postpartum. Boxplots depict median and inter-quartile range, overlaid with datapoints of individuals. Gray boxes represent pregnancy. Statistics performed by Wilcoxon paired test between trimesters 1 and 3, as well as trimester 3 and 3 months postpartum.

CD56^bright^ and CD56^dim^ NK cells constitute broad phenotypes of human NK cells. To shed light on the detailed NK cell phenotype in MS pregnancy, we next employed a data-driven approach to characterize NK cell phenotypes in depth during pregnancy in MS. Here, we measured 12 markers relevant for NK cell activation, maturation and migration in a second cohort of female MS patients which received a higher frequency of follow-ups and additional MRI scans postpartum (n = 8, MS cohort, Berlin, see **Table 1**). Importantly, the analysis in this cohort confirmed the broad shifts in NK cell phenotypes CD56^bright^ and CD56^dim^, with increased CD56^bright^ NK cell frequencies and diminished CD56^dim^ frequencies in the third trimester followed by reversal of these changes postpartum (see results of manual analysis in **Figures 2A, B**). Analysis of MRI scans showed an increase of T2-weighted lesion volume at six months postpartum, which coincided with the postpartum reduction of CD56^bright^ NK cells, whereas the number of lesions did not change (**Supplementary Figures 4A, B**).

**Figure 3:**
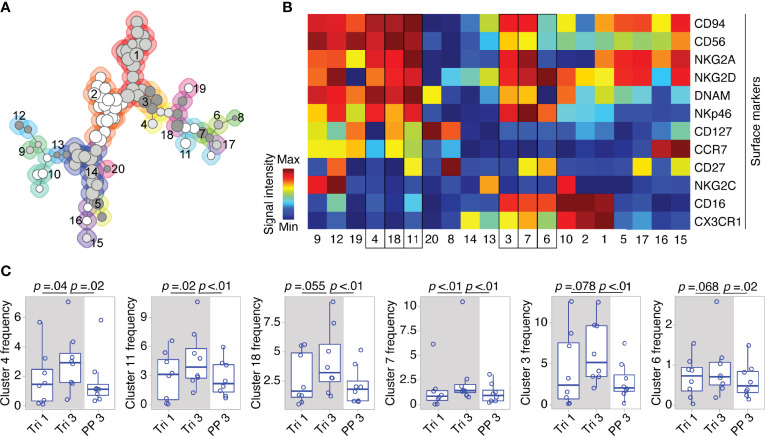
Clusters within CD56^bright^ and CD56^dim^ NK cell populations display dynamic shifts in MS pregnancy. In a detailed phenotypic analysis during and after pregnancy in MS patients (Berlin cohort), FlowSOM identified 20 distinct clusters among pre-gated Lin^-^ CD56^+^ NK cells. Clusters are numbered and indicated by shades of grey and differently coloured backgrounds (for details of FlowSOM see **Supplementary Figure 2** and Methods) **(A)**. Surface marker expression of each cluster is shown in the heatmap. For all markers except CD56, dark blue indicates negative expression and dark red indicates highly positive expression. For CD56, dark blue to green indicates dim expression (as all analyzed cells are CD56^+^) and dark red indicates bright expression **(B)**. Clusters with frequency changes throughout pregnancy and the postpartum period (six clusters with smallest p-values shown). Cluster frequency referred to as percentage of Lin^-^ CD56^+^ NK cells **(C)**. Cluster 3 and 7 contain CD56^bright^ and CD56^dim^ NK cells as indicated by a medium CD56 expression in the heatmap (yellow to orange). Mixed composition of cluster 3 and 7 has been additionally confirmed by manual gating. Boxplots depict median and inter-quartile range, overlaid with datapoints of individuals. The gray box represents pregnancy. Statistical analysis performed by Wilcoxon paired test between trimesters 1 and 3, as well as trimester 3 and 3 months postpartum.

On the detailed phenotypic level, computational analysis by means of the clustering algorithm FlowSOM identified 20 NK cell clusters with distinct marker expression profiles (see FlowSOM clusters in **Figures 3A, B**). Out of these, six clusters exhibited changes in their cell frequency over the course of pregnancy (six clusters with the smallest p-values, **Figure 3C**). To define the pregnancy-related phenotype of NK cells, we selected all clusters with significant frequency changes during and after pregnancy (see cluster 4, 11 and 7, **Figure 3C**), and characterized their marker expression.

Within the CD56^bright^ compartment, cluster 4 and 11 significantly increased from the first to the third trimester followed by a postpartum reduction (see **Figure 3C**). Taken together, cluster 4 and 11 formed more than half of the CD56^bright^ NK cells during pregnancy as they accounted for 6.8% of all CD56^+^ cells (versus 6.2% for the remaining CD56^bright^ clusters 18, 19, 12, and 9). Their phenotype was characterized by a dim expression of CD16, a receptor indicative for cell cytotoxicity, as well as by high expression levels of receptors that regulate cell activity, i.e., the activating receptors NKp46 and NKG2D, and the inhibitory receptor NKG2A (with NKG2D only being highly expressed on cluster 11; see cluster 4 and 11, **Figure 3B**). Due to this similar pattern of receptors for cell activity and cytotoxicity, cluster 4 and 11 can be regarded as one specific CD56^bright^ subset that might be of functional relevance during pregnancy (notwithstanding the differential expression CD127, CCR7 and CD27, see **Figure 3B**). Interestingly, cluster 7 with a mixed composition of CD56^bright^ and CD56^dim^ NK cells displayed high levels of NKp46, NKG2D and NKG2A comparable to the CD56^bright^ clusters 4 and 11 indicating that a minor fraction of CD56^dim^ NK cells shares the same expression pattern predominantly found on CD56^bright^ NK cells (see cluster 7, frequency changes of cluster 3 with a comparable marker expression did not reach statistical significance, **Figures 3B, C**). Reflecting the similar phenotype, the mixed cluster colocalized in the same MST region as the respective CD56^bright^ clusters (see **Figure 3A**). Out of 11 clusters containing only CD56^dim^ NK cells, no significant changes were observed during pregnancy and only one CD56^dim^ cluster (cluster 6) showed a significant postpartum decrease. Together, these high-dimensional phenotypic data from women with MS imply that the broad populations of CD56^bright^ and CD56^dim^ NK cells undergo even more fine-grade changes of their phenotype during pregnancy that may alter cell activity and cytotoxicity.

## Discussion

Our data confirmed that pregnancy in healthy women and MS induces a profound shift of NK cells toward the regulatory population of CD56^bright^ NK cells. Extending previous findings of pregnancy-related NK cell changes by exploratory high-dimensional phenotyping, we found a specific subset of CD56^bright^ cells that formed the predominant CD56^bright^ phenotype during pregnancy in MS, and was characterized by a dim expression of the cytotoxicity receptor CD16 and high levels of receptors that regulate cell activity, e.g., NKp46, NKG2D and NKG2A.

Although NK cells are discussed as potential regulators of autoimmune activity in MS (14, 16, 20), previous studies on pregnancy in MS have delivered conflicting results of how NK cell frequencies behave during this period of endogenous immune tolerance (24–27), likely due to incoherent study design. Here, we verified in two independent, longitudinal MS pregnancy cohorts that CD56^bright^ NK cell frequencies increase and CD56^dim^ NK cell frequencies decrease toward the third trimester followed by a reversal of these changes postpartum. Moreover, drawing on the so far largest longitudinal cohort of healthy pregnant women, we demonstrated a comparable pattern of a relative CD56^bright^ expansion and CD56^dim^ reduction for healthy pregnancy. Importantly, our data together with the results of others (24–26, 31, 32) imply that the observed NK cell shifts represent a general immune adaptation to pregnancy which can also be found in pregnant women with MS.

To yield deeper understanding of the role of NK cells during pregnancy in MS, analyses need to go beyond monitoring the prototypic NK cell phenotypes of CD56^bright^ and CD56^dim^. This becomes apparent in light of new data that reveal a far more complex phenotypic and functional diversity of human NK subsets that may be relevant for health and disease (33, 34). Here, we provided first in-depth profiling of the NK cell phenotype in MS pregnancy showing that the expansion of CD56^bright^ NK cells may be driven by a CD16^+^ NKp46^high^ NKG2D^high^ NKG2A^high^ subset. Additionally, a minor fraction of expanding CD56^dim^ NK cells also showed a comparable phenotype. As the sample for phenotyping was relatively small, our results should be regarded as exploratory. However, similar phenotypic NK data from healthy pregnancy (35) and pregnancy with type 1 diabetes (31) support the pattern of elevated expression of NKp46, NKG2D and NKG2A on NK cells.

The cell surface receptors we measured herein are indicative of cellular function, and our exploratory data on NK cell receptor expression should thus help to generate new hypotheses how NK cells may contribute to reinstall immune tolerance during pregnancy in MS. NK cell activity is tightly regulated through dynamic input from activating and inhibitory receptors (36). As inhibitory signals are necessary to render NK cells more responsive to increased activating stimuli (37), simultaneous upregulation of activating (e.g., NKp46 and NKG2D) and inhibitory receptors (e.g., NKG2A) during pregnancy could lead to increased NK cell responsiveness. Consequently, the CD56^bright^ NK cells observed in MS pregnancy might exert enhanced activity (i.e., cytokine production, or suppression of other immune cells through direct NK cytotoxicity). Along similar lines, the elevated CD16 expression on CD56^bright^ NK cells described here could indicate an enhanced cytotoxic capacity of CD56^bright^ cells in pregnant MS patients, given that this receptor for antibody-dependent cellular cytotoxicity (38) serves as an indicator of the overall cytotoxic potential of NK cells (39). Although we did not measure NK cell functions and activity of NK cells during healthy pregnancy has been a matter of debate (40, 41), NK cells from pregnant women might exhibit increased cytotoxicity upon adequate stimulation as lately shown in direct contact to influenza-infected PBMCs (35, 42).

On a mechanistic level, the specific CD56^bright^ phenotype of pregnancy in MS may lead to an increased regulatory NK cell activity, e.g., through enhanced cytotoxic suppression of activated T cells by CD56^bright^ NK cells, a cell interaction that was found impaired in MS (17, 18). NKp46, NKG2D and NKG2A are crucial for NK cell mediated suppression of autologous T cells (16). When T cells become activated, they increase their expression of NKG2D and NKG2A ligands, thereby allowing NK cells to control T cell activation through direct cytotoxicity (43, 44). In consequence, the specific CD56^bright^ subset we characterized herein might be able to suppress autoreactive T cells more efficiently, possibly contributing to the decreased inflammatory MS activity during pregnancy. Moreover, highly expanded T cell clones in MS patients were recently shown to contract during pregnancy (13), and NK cell mediated suppression could offer a potential explanation of these T cell repertoire changes. However, as MS patients exhibit increased levels of the inhibitory NKG2A ligand HLA-E (17, 45), it remains unknown how this aberrant expression may impact the actual NK cell activity in MS pregnancy. Another target of NK mediated tolerance induction could be dendritic cells (DC). NK cells shape the maturation of DCs through direct cytotoxicity that is dependent on NKp30, but also on NKp46 and NKG2A (46, 47), thus controlling antigen presentation to T cells which might constitute an alternative pathway how CD56^bright^ NK cells observed here limit autoreactive T cell responses.

Some limitations of our study should be considered. Although NK cell receptors are linked to cell function, we cannot infer functional consequences from the exploratory data on pregnancy-related NK phenotype modulations. It would thus be valuable to investigate NK cell functions in MS pregnancy. Such studies could also profit from exploring the relationship between NK cell characteristics and measures of MS disease activity (e.g., CNS lesions in MRI), an association that we could not analyze due to limited statistical power. With respect to the potential molecular drivers, it remains unclear whether NK cell changes during pregnancy represent specific immune adaptations that were evolutionary selected to sustain placental pregnancy [as compared to (5)], or represent an epiphenomenon of pregnancy-induced shifts in cytokines (48) and elevated levels of circulating hormones (49). Especially, hormones like estrogens, progesterone and cortisol have been intensely discussed to contribute to the amelioration of MS during pregnancy (8), however, their precise effect on NK cells is difficult to predict as various studies reported either a suppression (50–52) or stimulation (53, 54) of NK cell responses. Furthermore, studies in human subjects, especially in pregnant women, are inherently restricted to correlational observations, thus, precluding any causal links. However, as pregnancy leads to rapid and pronounced changes of disease activity in MS, it may constitute an informative research paradigm to unveil protective NK cell responses. Herein, we found a CD56^bright^ NK cell phenotype to be associated with clinical remission (e.g., pregnancy) that might lead to an increased regulatory NK cell activity during this period. In consequence, our findings further establish the relevant role of NK cells in MS pathogenesis, and sketch possible pathways for NK cell mediated modulation of autoimmunity during pregnancy.

## Data Availability Statement

The raw data supporting the conclusions of this article will be made available by the authors, without undue reservation.

## Ethics Statement

The studies involving human participants were reviewed and approved by Ethikkommission der Ärztekammer Hamburg; PV3558, PV3694; Ethikkommission der Charité – Universitätsmedizin Berlin, EA1/173/13. The patients/participants provided their written informed consent to participate in this study.

## Author Contributions

SG conceived the study. SG, AD, MF, PA, ET, and FP acquired funding. AD, CH, NS, FP, and SG planned and conducted recruitment, follow-up, and sample collection. SG, CD, AW, and MS designed the experiments. AW conducted experiments. AW, CR, SS, and SG analyzed the data. AW, CR, SS, and SG interpreted the data. CR, AW, SS, and SG designed figures and tables. AW, CR, and SG wrote the manuscript. All authors critically revised the manuscript for intellectual content. All authors contributed to the article and approved the submitted version.

## Funding

This work was supported by the Deutsche Forschungsgemeinschaft (grants GO1357/8-1, GO1357/8-2, FR1720/8-1, FR1720/8-2 to SG and MF, DI2103/2-2 to AD, AR232/25-2 to PA., and KFO296/S1 project to AD and ET as part of the KFO296 Fetomaternal Immune Cross-Talk). CR was supported by the Forschungsförderungsfond der Medizinischen Fakultät, Universitätsklinikum Hamburg-Eppendorf. A-RL was supported by fellowships from the European Federation of Immunological Societies (EFIS), the German Academic Exchange Service (DAAD), the Multiple Sclerosis International Federation (MFIS), and the UKE Hamburg Southeast Europe Cooperation.

## Conflict of Interest

The authors declare that the research was conducted in the absence of any commercial or financial relationships that could be construed as a potential conflict of interest.

## Publisher’s Note

All claims expressed in this article are solely those of the authors and do not necessarily represent those of their affiliated organizations, or those of the publisher, the editors and the reviewers. Any product that may be evaluated in this article, or claim that may be made by its manufacturer, is not guaranteed or endorsed by the publisher.
