# Supplementary material for: Alterations of NK Cell Phenotype During Pregnancy in Multiple Sclerosis
Source: Front Immunol. 2022 Jul 4;13:907994. doi: 10.3389/fimmu.2022.907994 (PMC9289470; doi:10.3389/fimmu.2022.907994)
Supplement: Supplementary file 1 [file DataSheet_1.docx]

Supplementary Material

# Supplementary Figures


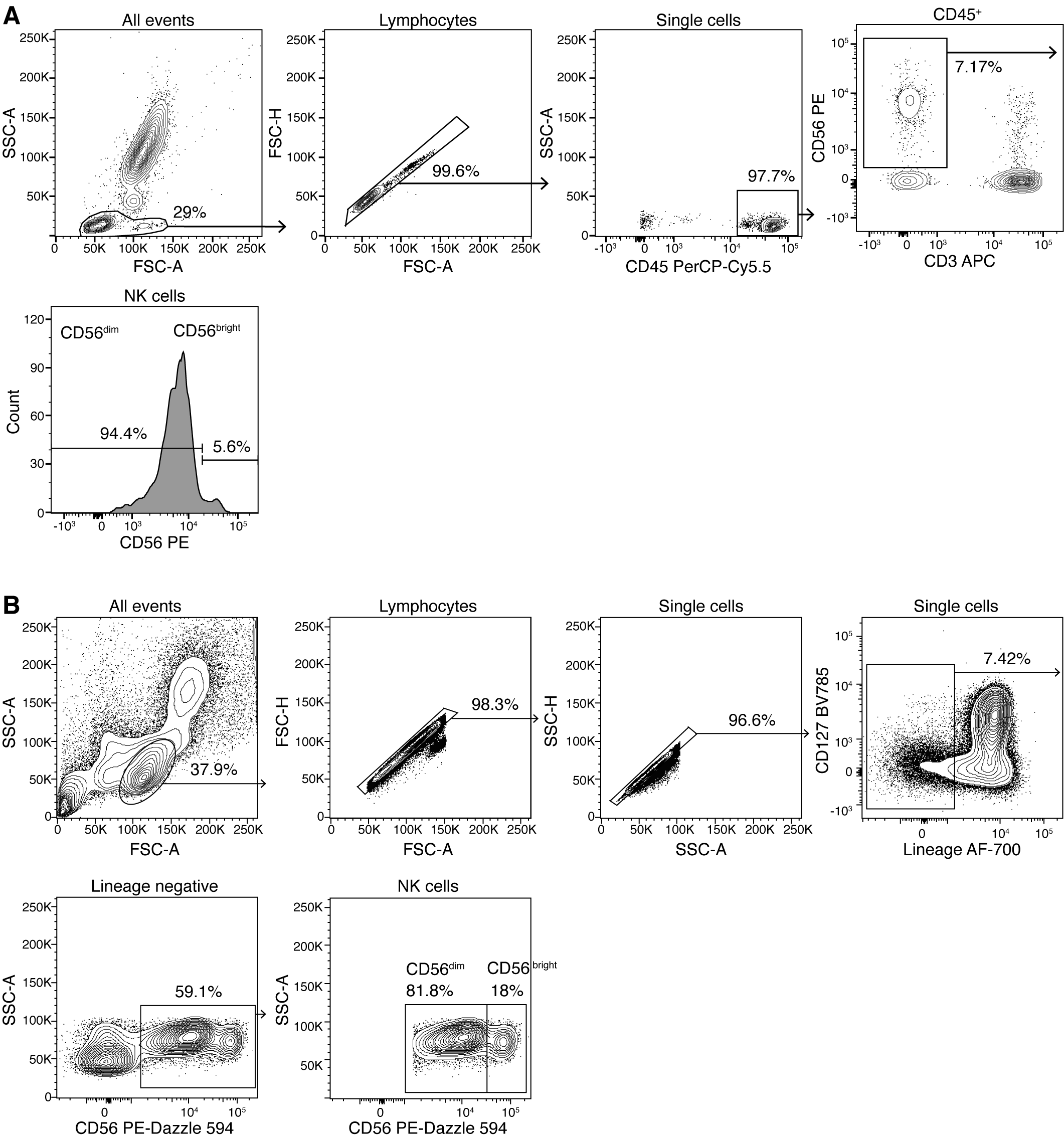


**Supplementary Figure 1. Manual gating strategy of flow cytometry data.** Healthy and MS cohorts from Hamburg were gated on lymphocytes, single cells, CD45^+^ leukocytes, CD56^+^ CD3^-^ and then divided into CD56^bright^ and CD56^dim^ NK populations (**A**). Data from the Berlin MS cohort were gated for lymphocytes, single cells, Lineage negative (Lin^-^) cells excluding CD3^+^/CD19^+^/CD20^+^/CD14^+^ cells. Subsequently, CD56^+^ NK cells were selected and divided into the populations CD56^bright^ and CD56^dim^ (**B**).


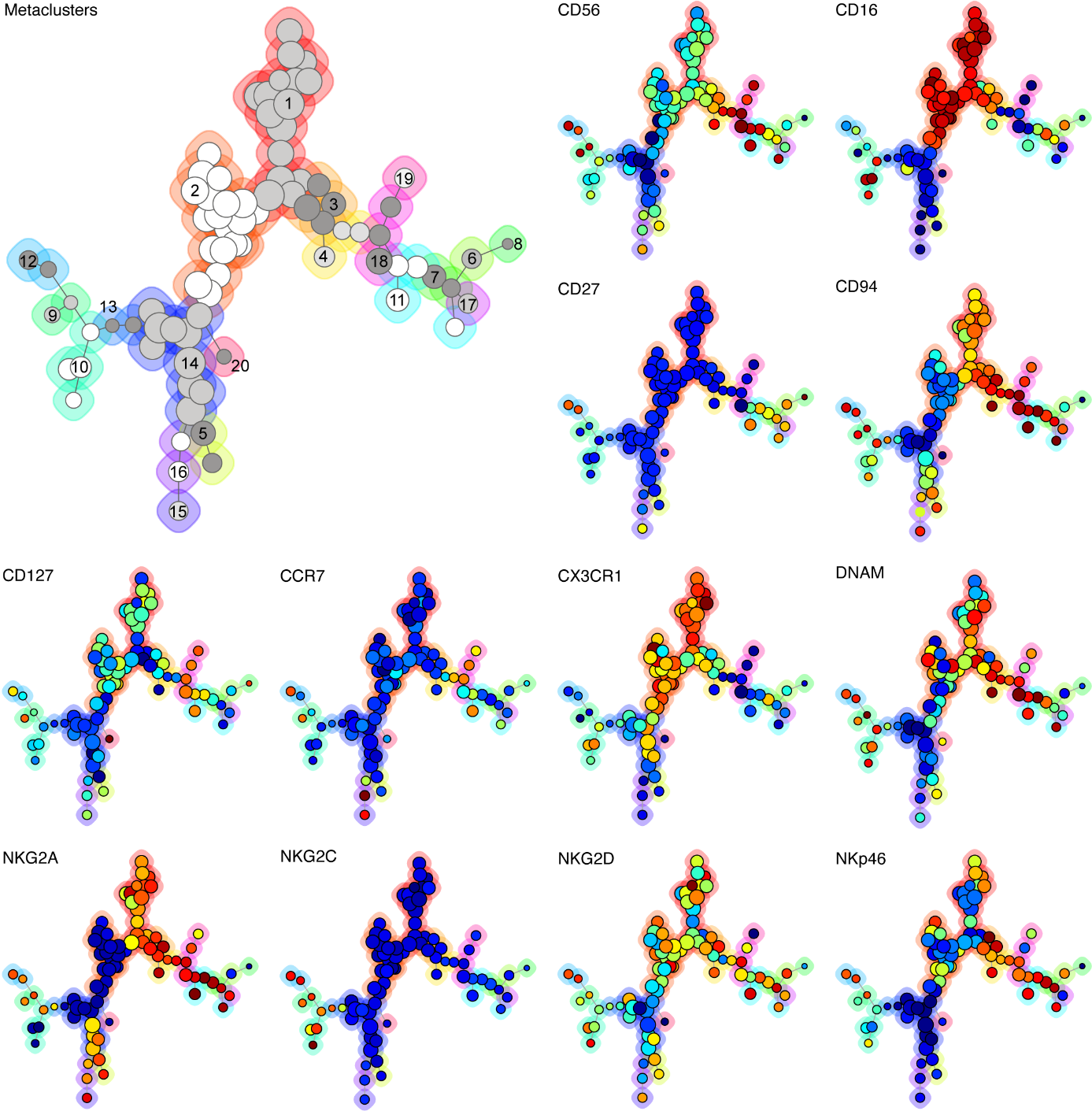


**Supplementary Figure 2. Marker expression of FlowSOM clusters.** Using FlowSOM, pre-gated Lin^-^ CD56^+^ NK cells were clustered based on 12 NK cell markers. The individual nodes of the minimal spanning tree (MST) correspond to the individual clusters of phenotypically similar cells (81 clusters in total). Individual clusters are grouped in 20 metaclusters that are indicated by numbers, shades of grey and differently coloured backgrounds (see annotation of metaclusters). In the results section, metaclusters are simply referred to as clusters. The size of the nodes shows the number of cells in an individual cluster (the larger, the more cells). Clusters that are located in close proximity within the MST contain cells with similar marker expression. The colour of the clusters represents the median fluorescence intensity of the respective marker (minimum, blue; maximum, red): For all markers except CD56, dark blue indicates negative expression and dark red indicates highly positive expression. For CD56, dark blue indicates very dim expression (as all analyzed cells are CD56^+^) and dark red indicates bright expression.

**
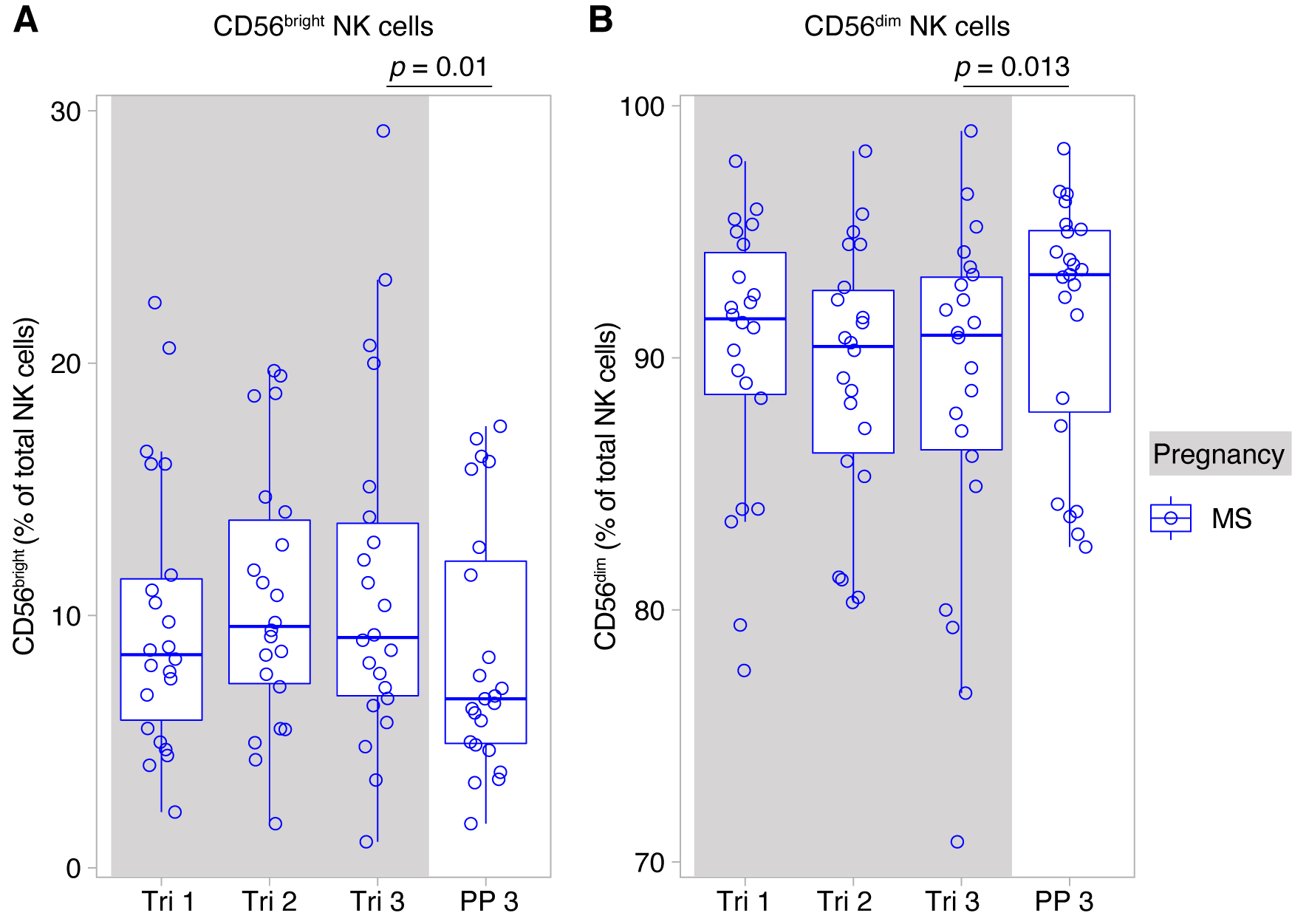
**

**Supplementary Figure 3. Pregnancy-induced NK cell shifts reverse after pregnancy.** Frequency of CD56^bright^ NK cells (**A**) and CD56^dim^ NK cells (**B**) in pregnant MS patients (MS, Hamburg cohort) shown for each trimester of gestation and 3 months postpartum. Boxplots depict median and inter-quartile range, overlaid with datapoints of individuals. Gray boxes represent pregnancy. Statistics performed by Wilcoxon paired test.

**
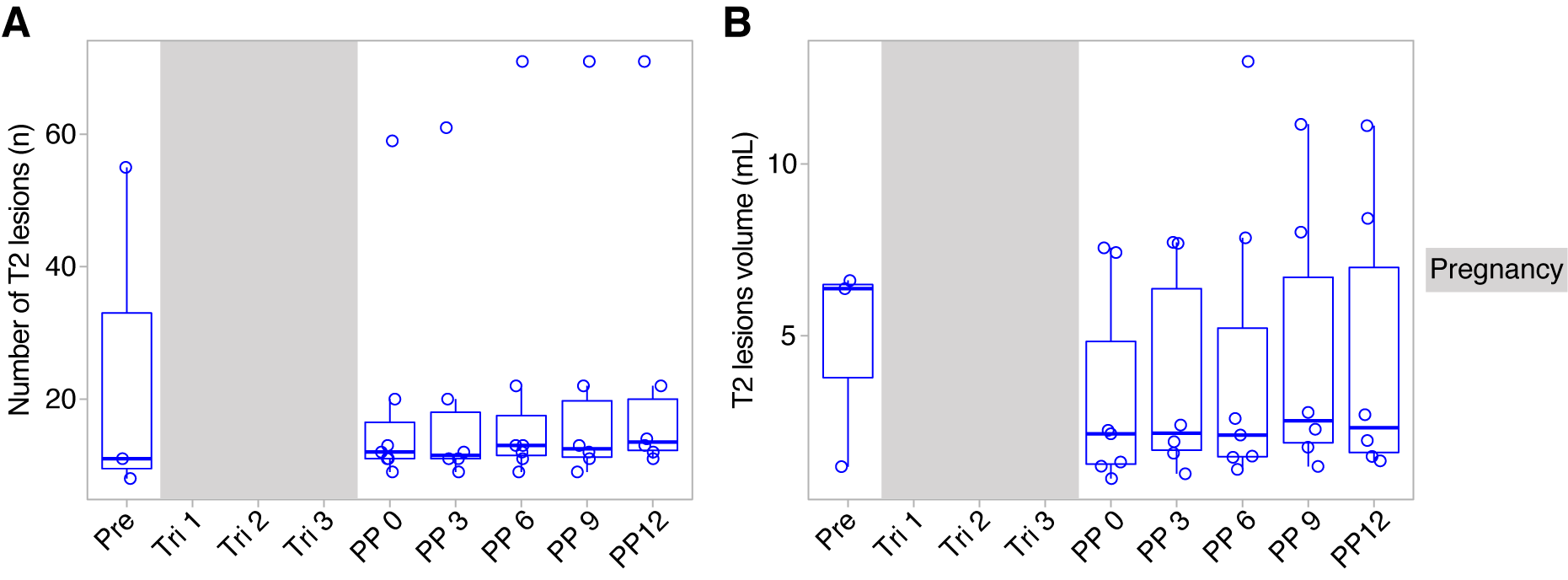
**

**Supplementary Figure 4. Neuroinflammatory activity in the Berlin MS cohort.** Neuroinflammatory activity is shown as absolute number of T2 lesions (**A**) and absolute lesion volume (**B**). Boxplots depict median and inter-quartile range, overlaid with datapoints of individuals. Gray boxes represent pregnancy.
